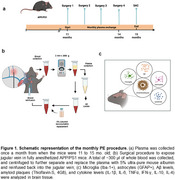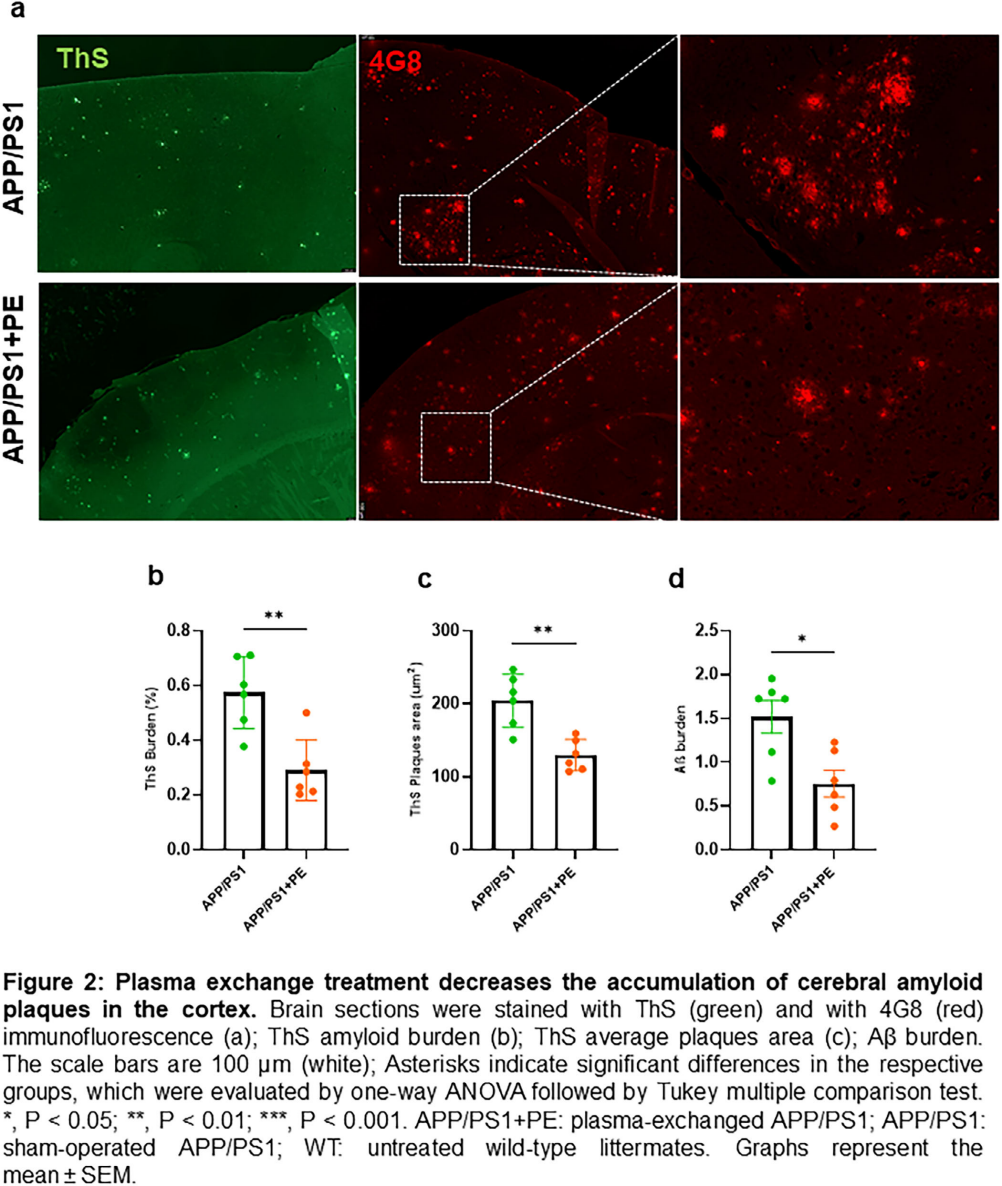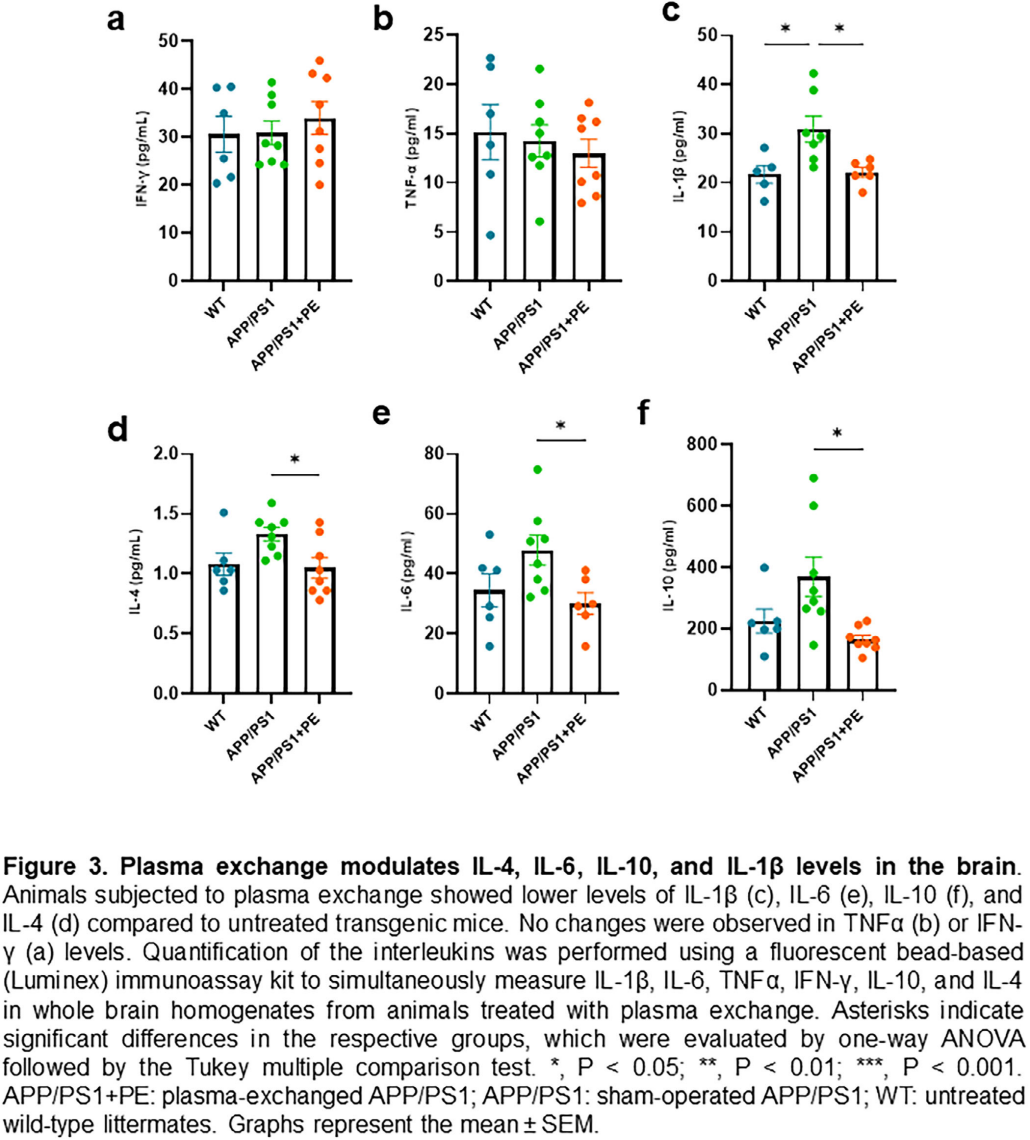# Reduction of amyloid plaques and modulation of neuroinflammation in APP/PS1 mice at advanced stages of Alzheimer's disease by Plasma exchange with albumin replacement.

**DOI:** 10.1002/alz70859_100925

**Published:** 2025-12-25

**Authors:** Suelyn Koerich, Santiago Ramirez, Natalia Astudillo, Gabriela Delevati Colpo, Claudio Soto

**Affiliations:** ^1^ The University of Texas Health Science Center at Houston, Houston, TX USA

## Abstract

**Background:**

Alzheimer’s disease (AD) is the leading cause of dementia, marked by the accumulation of extracellular amyloid plaques, intraneuronal neurofibrillary tangles, and neuroinflammation, all contributing to cognitive decline. Although disease‐modifying therapies targeting amyloid plaques show promise, eligibility for these treatments is limited, and studies suggest that the risks may outweigh the potential benefits for many patients. Recent preclinical studies suggest that peripheral approaches may influence amyloid (Aβ) and tau deposition, as well as neuroinflammation in the brain. Plasma exchange (PE), where plasma is replaced with albumin, has emerged as a potential treatment for AD. This study investigates the effects of PE on Aβ pathology and neuroinflammation in APP/PS1 mice at an advanced stage of amyloid pathology.

**Methods:**

Four PE procedures were performed monthly starting at 11 months of age. At 15 months, all animals were euthanized, and brains were processed for histological analysis. Amyloid deposits were assessed with Thioflavin‐S (ThS) and 4G8 immunostaining in the cortex and hippocampus. ELISA was used to measure soluble and insoluble Aβ_1–40_ and Aβ_1–42_ in brain homogenates and plasma. Inflammatory responses were quantified using a Luminex bead‐based immunoassay for cytokines (IL‐1β, IL‐6, TNFα, IFN‐γ, IL‐10, IL‐4) in whole‐brain homogenates and plasma. Iba‐1 and GFAP immunostaining assessed microglial and astrocyte areas.

**Results:**

PE‐treated animals showed significant reductions in amyloid plaque number and area in the cortex compared to untreated APP/PS1 mice. Additionally, Iba‐1+ and GFAP+ areas were significantly smaller in PE‐treated animals. Biochemical analysis confirmed reductions in insoluble Aβ1–40 and Aβ_1–42_ levels in the brain of PE‐treated animals. PE also modulated the inflammatory response, decreasing IL‐1β, IL‐6, IL‐10, and IL‐4 in the brain compared to untreated mice.

**Conclusions:**

These findings suggest that PE may reduce amyloid burden and modulate inflammation in the brain. An increase in plasma Aβ_1–40_ and Aβ_1–42_ after PE suggests Aβ movement from the brain to the blood. Further studies are needed to elucidate the mechanisms underlying Aβ clearance and inflammation modulation.